# Birth weight and risk of ischemic heart disease: A Mendelian randomization study

**DOI:** 10.1038/srep38420

**Published:** 2016-12-07

**Authors:** Shiu Lun Au Yeung, Shi Lin Lin, Albert Martin Li, C. Mary Schooling

**Affiliations:** 1 School of Public Health, Li Ka Shing Faculty of Medicine, The University of Hong Kong, Hong Kong SAR, China; 2 Department of Pediatrics, Faculty of Medicine, The Chinese University of Hong Kong, Hong Kong SAR, China; 3 City University of New York, Graduate School of Public Health and Health Policy, New York, NY, USA

## Abstract

Low birth weight is a risk factor for cardiovascular disease. However, the association could be confounded by many factors. We used Mendelian randomization to clarify the role of birth weight in ischemic heart disease (IHD) and lipids. We used all 7 single nucleotide polymorphisms (SNPs) independently contributing to birth weight at genome wide significance (p < 5 × 10^−8^) in separate sample instrumental variable analysis to estimate the effect of birth weight on IHD using the CARDIoGRAMplusC4D 1000 Genomes based GWAS case (n = 60,801)-control (n = 123,504) study and on lipids using GLGC (n = 188,577). Higher genetically predicted birth weight was associated with lower risk of IHD (odds ratio (OR) 0.96 per 100 grams, 95% confidence interval (CI) 0.93 to 0.99), but the association was not robust to sensitivity analyses excluding SNPs related to height or use of weighted median methods. Genetically predicted birth weight was not associated with low density lipoprotein cholesterol or triglycerides, but was associated with lower high density lipoprotein cholesterol (−0.014 standard deviation, 95% CI −0.027 to −0.0005) and the association was more robust to the sensitivity analyses. Our study does not show strong evidence for an effect of birth weight on IHD and lipids.

Following seminal observations from the 1980 s onwards showing lower birth weight associated with higher blood pressure^1^, ischemic heart disease (IHD)^2^, and diabetes^3^, and intensive investigation over the last quarter of a century, low birth weight has been classified by the World Health Organization as a risk factor for cardiovascular disease^4^. Nevertheless controversy has arisen as to the relevant intervention to improve population health because of the analytic challenges of isolating the effect of one of many linked factors using observational studies, and the uncertainty as to whether the causal factor is birth weight or some aspect of inter- and intra-generational environmental conditions, maternal experiences or genetics. IHD rates do not obviously fall with the improved living conditions that might enable higher birth weight^5^. Randomized controlled trials (RCTs) designed to increase birth weight have had mixed results^6^,^7^, and a trial of birth weight would require long-term follow up from before birth or even conception to at least mid-adulthood to reliably assess effects on IHD. Nevertheless, understanding the role of birth weight in cardiovascular disease is important from a public health perspective given low birth weight is prevalent in the low and middle income countries where an epidemic of cardiovascular disease is currently emerging^8^.

Instrumental variable analyses, using an external variable as an instrument instead of assuming no unmeasured confounding, provides an alternative means of assessing the role of birth weight in health. To date, studies using instruments, such as birth rank^9^, or twin status^10^, have suggested little association of birth weight with blood pressure or cardiovascular disease risk factors but birth rank and twin status are of uncertain validity as instruments for birth weight. In contrast, comparing risk of disease in people with genetically higher birth weight, i.e., using instrument variable analysis with genetic instruments, (Mendelian randomization (MR)) provides an increasingly popular means of obtaining unconfounded estimates of potentially confounded associations, because genetic determinants of birth weight are randomly allocated at conception, analogous to the randomization in randomized controlled trials, and hence allow estimation of the causal effect of birth weight on health, provided that the relevant assumptions are fulfilled. Recently, a Mendelian randomization study confirmed observations of an inverse relation of birth weight with type 2 diabetes^11^. However, to date, no MR study has examined the role of birth weight in cardiovascular disease. Here we used a similar approach to examine the causal role of birth weight in IHD and lipids.We used a genome wide association study (GWAS) to obtain genetically determined birth weight^12^, and to reduce the likelihood of false negatives, very large case-control studies of IHD and myocardial infarction (MI) (CARDIoGRAMplusC4D 1000 Genomes based GWAS)^13^,^14^,^15^,^16^, and large studies of lipids^17^, to assess the role of genetically predicted birth weight.

## Results

Based on the largest GWAS of birth weight to date, 7 uncorrelated SNPs (rs724577 (*LCORL*), rs900400 (*CCNL1*), rs1042725 (*HMGA2*), rs1801253 (*ADRB1*), rs4432842 (*5q11.2*), rs6931514 (*CDKAL1*), rs9883204 (*ADCY5)* reaching genome wide significance for birth weight were identified^12^. Appendix 1 summarizes the information extracted for each SNP for CAD/MI and MI. Five SNPs had potentially pleiotropic effects. rs724577 and rs1042725 are associated with height. rs1801253 is associated with blood pressure which is a known cause of CAD/MI. rs6931514 (*CDKAL1*) and rs9883204 (*ADCY5)* are associated with type 2 diabetes. Whether any association of these 5 SNPs with IHD operates solely via birth weight or instead directly via height, blood pressure or diabetes is not known, so estimates are provided with and without these SNPs.

**Figure 1:**
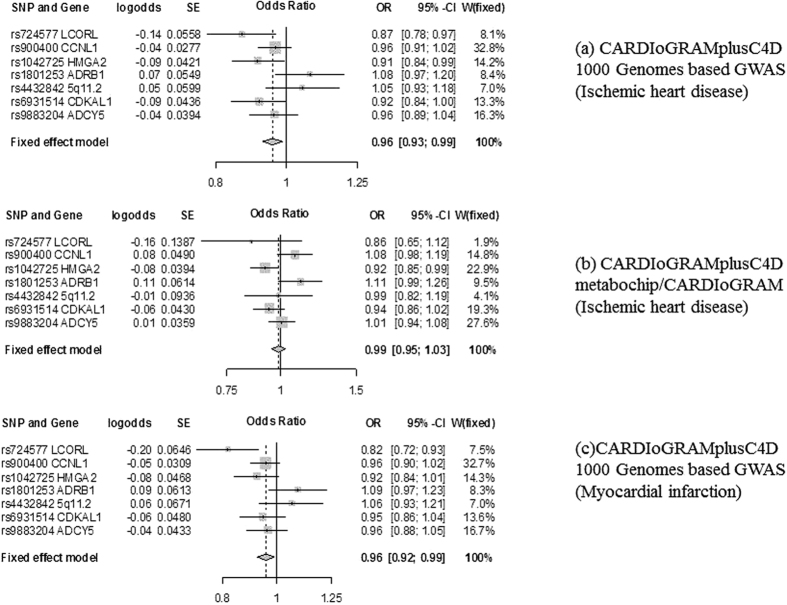
Forest plots for the Mendelian randomization design of birth weight^12^ and ischemic heart disease and myocardial infarction using CARDIoGRAMplusC4D 1000 Genomes based GWAS, and CARDIoGRAMplusC4D metabochip/CARDIoGRAM^13^,^14^,^15^,^16^.

Using all 7 SNPs and IVW genetically predicted birth weight had small inverse associations with CAD/MI (OR 0.96 per 100 grams, 95% CI 0.93 to 0.99) and MI (OR 0.96 per 100 grams, 95% CI 0.92 to 0.99) using CARDIoGRAMplusC4D 1000 Genomes based GWAS, as shown in Table 1. Figure 1a and c show the SNP-specific estimates. No association was evident using the CARDioGRAMplusC4D metabochip/CARDIoGRAM GWAS, as shown in Table 1. Figure 1b shows the SNP-specific estimates. There was no evidence for directional horizontal pleiotropy according to the MR-Egger regression intercept (p value > 0.54 for the analyses on IHD and MI using 7 SNPs, consistent with the symmetries in associated funnel plots (Appendix 2a to c)). Comparing Fig. 1a and b the difference between the two estimates for CAD/MI is due to directionally different estimates for rs900400 and a stronger association of rs4432842 with CAD/MI in CARDIoGRAMplusC4D 1000 Genomes based GWAS. The inverse association in CARDIoGRAMplusC4D 1000 Genomes based GWAS was not robust to methodological choices, such as use of a weighted median method or exclusion of potentially pleiotropic SNPs. For example, after removal of the two potentially pleiotropic SNPs associated with height, little association of lower genetically predicted birth weight with CAD/MI or MI was evident.

**Figure 2:**
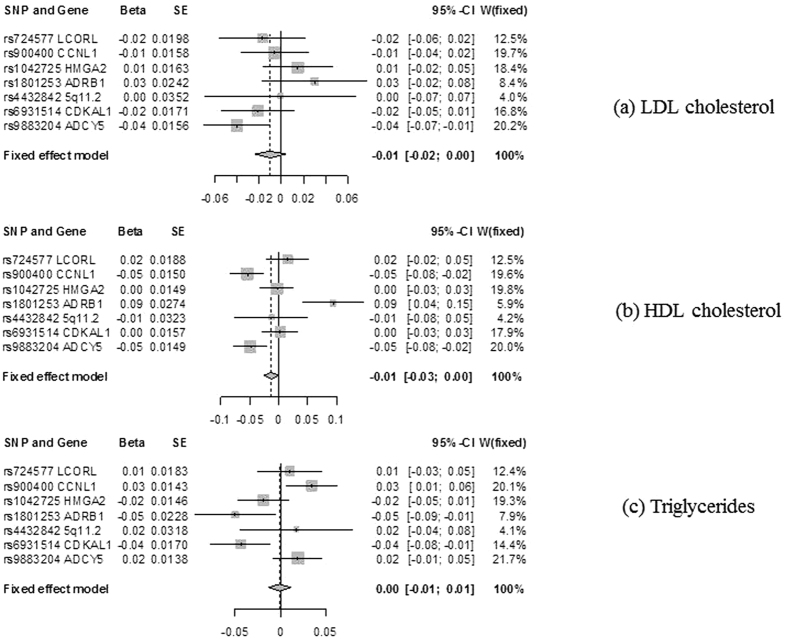
Forest plots for the Mendelian randomization design of birth weight^12^ and LDL cholesterol, HDL cholesterol, and triglycerides using Global Lipids Genetic Consortium^17^.

Table 2 shows that genetically predicted birth weight was not associated with LDL-cholesterol (Fig. 2a) or triglycerides (Fig. 2c). There was no evidence for directional horizontal pleiotropy according to the MR-Egger regression intercept (p value > 0.20 for LDL cholesterol and triglycerides using 7 SNPs, consistent with the symmetries in associated funnel plots (Appendix 2d and f)). Higher genetically predicted birth weight showed some indications of being associated with slightly lower HDL-cholesterol (Fig. 2b) where there was some evidence for directional horizontal pleiotropy (p value: 0.046 for HDL cholesterol using 7 SNPs). The corresponding funnel plot (Appendix 2e) also showed more extreme outlier SNPs compared to the other plots. However, this association was robust to the exclusion of the 2 SNPs associated with height and use of a weighted median estimator in the analysis using 5 SNPs.

## Discussion

This novel study using Mendelian randomization, a potentially less confounded study design, is consistent with previous meta-analysis of observational studies suggesting that lower birth weight may be associated with a slightly higher risk of IHD^18^,^19^. Our study also adds by showing a similar estimate of the effect of lower birth weight on MI. However, the estimates for IHD and MI were not robust to the exclusion of potentially pleiotropic SNPs affecting height or to the use of more conservative methods. Our findings are also largely consistent with meta-analysis of observational studies suggesting birth weight has little association with lipids^20^, although our study raises, for the first time, the possibility that higher birth weight is associated with lower HDL-cholesterol.

We used separate sample instrumental variable analysis with genetic instruments which is less susceptible to residual confounding than observational studies. However, limitations exist. First, Mendelian randomization has stringent assumptions concerning the validity of the genetic instruments, i.e., the genetic variants predicting birth weight. These assumptions include that the genetic variants reliably predict birth weight, no confounders of the genetic variants and the outcomes exist and all the effects of the genetic variants on each outcome are only via the exposure, i.e., birth weight. The genetic predictors of birth weight strongly predicted birth weight at genome wide significance, which reduces the risk of false positives. Confounding of the relation between genetic variants and the outcomes is unlikely because we used separate samples for birth weight and the outcomes which reduces the risk of chance associations generated by any common underlying data structure. Overlaps between the samples used to obtain genetic predictors of birth weight and their effects were not substantial. The samples used were largely of people of European descent and included genomic control to avoid any hidden genetic associations or confounding by population stratification. However, the estimates for IHD were not robust to the use of different cases-control studies. Use of CARDIoGRAMplusC4D 1000 Genomes based GWAS with a larger number of cases and controls but with more non-Europeans gave an inverse association of genetically predicted birth weight with IHD while CARDIoGRAMplusC4D metabochip/CARDIoGRAM GWAS with fewer cases for some SNPs, such as rs900400, and fewer non-Europeans gave a null association. Similarly, the estimates were not robust to different methodological approaches, such as use of a weighted median estimation, which may be more robust to inclusion of invalid instruments. We also assumed homogeneity and linearity between birth weight and the outcomes with no effect modification^21^. Overall given these assumptions, the estimate is best interpreted as indicating direction rather than the precise size of the effect^22^. Second, we were unable to check whether the associations varied by sex, when age-standardized IHD rates are much higher in men than women, HDL-cholesterol is lower in men than women, and birth weight may have different associations with lipids by sex^23^. Finally, we only considered fetal, not maternal, genetics, so the effects of specifically maternally driven determinants of birth weight, such as maternal smoking, might be biased towards the null as mother and fetus only share half their genetic make-up. As such, these findings require replication in different populations and settings.

The genetic predictors of birth weight are in genes associated with height (*LCORL* and *HMGA2*)^12^, type 2 diabetes (*CDKAL1* and *ADCY5*)^12^, blood pressure (*ADRB1*)^12^, possibly epigenetic processes (*CCNL1)*^24^, and 5q11.2 whose function is not clearly understood. As such, we cannot exclude the possibility that these genetic predictors of birth weight affect IHD and/or lipids directly rather than only through birth weight. Most notably, maternal height determines birth weight^25^ and height determines IHD^26^, and our estimates for IHD and MI were not robust to the exclusion of genetic variants affecting height (Table 1).

Our findings also do not exclude a small inverse association of birth weight with IHD of a fairly similar magnitude to those found in meta-analysis of observational studies^18^,^19^, although Mendelian randomizations estimates may be inflated by imprecise measurement of the exposure^27^. On the other hand, we cannot exclude no association but twins usually have lower birth weight but not a different risk of cardiovascular disease from their siblings^28^. Notably, our findings for the association of birth weight with IHD and MI appear to be less marked that those recently reported from a Mendelian randomizations study of birth weight and diabetes using the same genetic variants^11^. Diabetes is a strong predictor of CAD/MI, however it is becoming increasingly evident that factors do not always have the same effect on diabetes and CAD/MI, such as lipids or statins^29^. Our findings for lipids are fairly consistent with well-conducted observational studies^30^ and instrumental variable analysis using birth rank as an instrument for birth weight^9^, which found no association of birth weight with HDL and triglycerides. Our findings do not exclude the possibility of a small association of higher birth weight with lower HDL-cholesterol. Such an association, if it exists, might have been obscured by negative confounding by socio-economic position in previous observational studies^30^ and by lack of power in studies using instrumental variable analysis^9^.

The role of birth weight in health has been intensively investigated for over 25 years. Taking together evidence concerning the role of birth weight from this study and the previous Mendelian randomization on diabetes^11^, suggests birth weight may play a more protective role in diabetes than IHD, while raising the possibility of an adverse effect on specifically HDL-cholesterol. Better understanding of the determinants and consequences of birth weight should inform interventions to change birth weight or to compensate for any effects of lower birth weight, given higher birth weight may raise the risk of cancer. These considerations are particularly relevant for developing countries where the optimal growth pattern is unknown but interventions are currently focused on birth weight and growth.

## Methods

### Genetic predictors of birth weight

Genetically predicted birth weight (z-score) was obtained from all single nucleotide polymorphisms (SNPs) strongly associated (at genome wide significance p-value < 5 × 10^−8^) using the largest published GWAS of birth weight^12^. For ease of interpretation, z-scores were converted to 100 gram units (assuming the standard deviation of birth weight (z-score) is 484 grams). Correlations between the selected SNPs (linkage disequilibrium) were assessed from SNP Annotation and Proxy Search (http://www.broad.mit.edu/mpg/snap/ldsearchpw.php). To rule out the possibility of violation of the instrumental variable exclusion restriction assumption by pleiotropic SNPs directly affecting the CAD/MI or lipids other than via birth weight, we also checked for pleiotropic effects of each of the selected SNPs on these outcomes from a comprehensive genotype to phenotype cross-reference, Ensembl (http://www.ensembl.org/index.html), which contains information on any traits strongly associated with any given SNP.

### Genetic predictors of coronary artery disease/myocardial infarction and lipids

Data on coronary artery disease (CAD)/myocardial infarction (MI) have been contributed by CARDIoGRAMPLUSC4D investigators and have been downloaded from www.CARDIOGRAMPLUSC4D.ORG^13^,^14^,^15^,^16^. CARDIoGRAMplusC4D 1000 Genomes-based GWAS is a meta-analysis of GWAS of CAD case-control studies of people of mainly European, South Asian, and East Asian, descent imputed using the 1000 Genomes phase 1 v3 training set with 38 million variants. The study interrogated 9.4 million variants and included 60,801 IHD cases (~42,560 MI cases) and 123,504 controls with separate estimates for IHD and MI^16^. CARDIoGRAMplusC4D Metabochip is a two stage meta-analysis of Metabochip and GWAS of European and South Asian descent including 63,746 IHD cases and 130,681 controls. This study only directly genotyped non-monomorphic SNPs on the Illumina metabochip (only ~72,000 SNPs) whereas CARDIoGRAM GWAS is a subset of 22,233 IHD cases and 64,762 controls of European descent imputed to HapMap 2 (~2.5 million SNPs)^13^,^14^,^15^. From these case-control studies we obtained the association of each SNP with CAD/MI and where available with MI. Genetic associations with inverse normal transformed effect sizes of high density lipoprotein (HDL) cholesterol, low density lipoprotein (LDL) cholesterol and triglycerides have been contributed by Global Lipids Genetics Consortium (GLGC) investigators and have been downloaded from http://csg.sph.umich.edu/abecasis/public/lipids2013/ 17, which relates to people of European ancestry (n = 188,577)^17^.

### Statistical analyses

Estimates of the effect of genetically predicted birth weight on CAD/MI, MI and lipids were obtained from separate sample instrumental variable analysis by combining the SNP-specific Wald estimates using inverse variance weighting (IVW) with fixed effects^31^ with Fieller’s theorem used to obtain the variance of each Wald estimate. A Wald estimate gives the population average effect of a marginal model when the proportion of variance in the exposure explained by the genetic variants is low^22^. From the analyses we reported the odds ratio (OR) for IHD and MI and the mean difference for lipids with 95% confidence interval (CI).

### Sensitivity analyses

We conducted three sensitivity analyses to assess whether the estimates were robust to methodological choices. First, we repeated the analyses excluding SNPs that might be associated with the specific outcome other than via birth weight (i.e., have pleiotropic effects). Second, we repeated the analysis including SNPs that did not meet GWAS significance for birth weight, but were strongly associated with birth weight (p-value < 10^−5^) to rule out bias due to inclusion of insufficient instruments. Third, we examined the intercept in the analysis with all 7 SNPs using MR-Egger regression, which provides a test for directional horizontal pleiotropy^32^. We also provided the corresponding funnel plot for visual inspection of potential directional horizontal pleiotropy as the MR-Egger regression intercept test may be underpowered due to the small number of SNPs. Lastly, we repeated the analysis using weighted median estimation, which may generate correct parameter estimation as long as invalid genetic instruments contribute no more than 50% of the information in the estimation of the association^33^.

All statistical analyses were conducted using Stata version 13.1 (StataCorp LP, College Station, TX) and R version 3.2.3 (R Foundation for Statistical Computing, Vienna, Austria). This study only used publicly available data and hence no ethical approval from Institutional Review Board was required.

## Additional Information

**How to cite this article**: Au Yeung S. L. *et al*. Birth weight and risk of ischemic heart disease: A Mendelian randomization study. *Sci. Rep.* **6**, 38420; doi: 10.1038/srep38420 (2016).

**Publisher’s note:** Springer Nature remains neutral with regard to jurisdictional claims in published maps and institutional affiliations.

## Supplementary Material

Supplementary Information

## Figures and Tables

**Table 1:** Estimates of the effect of genetically predicted birth weight (per 100 gram)^12^ on ischemic heart disease (IHD) and myocardial infarction (MI)^13^,^14^,^15^,^16^ obtained from Mendelian randomization using different data sources, different methodological approaches and different exclusions for pleiotropy.

Disease	Data source	Method	7 SNPs		5 SNPs^a^		4 SNPs^b^		2 SNPs^c^,^d^		10 SNPs^e^	
			Odds ratio	95% CI	Odds ratio	95% CI	Odds ratio	95% CI	Odds ratio	95% CI	Odds ratio	95% CI
IHD	CARDIoGRAMplusC4D 1000 Genomes based GWAS	IVW	0.96	0.93 to 0.99	0.97	0.94 to 1.01	0.96	0.93 to 1.00	0.98	0.93 to 1.03	0.96	0.93 to 0.99
		WM	0.96	0.92 to 1.00	0.96	0.92 to 1.01	0.96	0.92 to 1.01	N/A		0.96	0.92 to 1.00
	CARDIoGRAMplusC4D metabochip/CARDIoGRAM	IVW	0.99	0.95 to 1.03	1.02	0.97 to 1.06	1.00	0.96 to 1.05	1.06	0.97 to 1.16	0.99	0.96 to 1.03
		WM	0.99	0.94 to 1.05	1.01	0.95 to 1.07	1.00	0.94 to 1.06	N/A		0.99	0.94 to 1.04
MI	CARDIoGRAMplusC4D 1000 Genomes based GWAS	IVW	0.96	0.92 to 0.99	0.98	0.94 to 1.02	0.97	0.93 to 1.01	0.97	0.92 to 1.03	0.96	0.93 to 0.99
		WM	0.95	0.91 to 1.00	0.96	0.91 to 1.00	0.96	0.91 to 1.00	N/A		0.95	0.91 to 0.99

^a^Excluding SNPs related to height (rs724577(*LCORL*) and rs1042725 (*HMGA2*)).

^b^Excluding SNPs related to height (rs724577(*LCORL*) and rs1042725 (*HMGA2*)) and blood pressure (rs1801253 (*ADRB1*)).

^c^Excluding SNPs related to height (rs724577(*LCORL*) and rs1042725 (*HMGA2*)), blood pressure (rs1801253 (*ADRB1*)) and type 2 diabetes (rs6931514 (*CDKAL1*), rs9883204 (*ADCY5)*).

^d^Weighted median method not available as one SNP contributed more than 50% of the information in the analysis.

^e^Including SNPs which did not reach genome wide significance for birth weight but p value <10^−5^ (rs5415 (*SLC2A4*); rs5758511 (*CENPM*); and rs7780752 (*CALCR*)).

IVW: Inverse variance weighting method; WM: Weighted median method.

**Table 2:** Estimates of the effect of genetically predicted birth weight (per 100 gram)^12^ with lipids^17^ obtained from Mendelian randomization using different methodological approaches and different exclusions for pleiotropy.

Lipid traits	Source	Method	7 SNPs		5 SNPs^a^		10 SNPs^b^	
			Beta	95% CI	Beta	95% CI	Beta	95% CI
LDL cholesterol (SD)	Global Lipids Genetics Consortium	IVW	−0.0096	−0.023 to 0.004	−0.015	−0.031 to 0.002	−0.012	−0.026 to 0.0008
		WM	−0.013	−0.032 to 0.006	−0.017	−0.038 to 0.005	−0.016	−0.035 to 0.003
HDL cholesterol (SD)	Global Lipids Genetics Consortium	IVW	−0.014	−0.027 to −0.0005	−0.022	−0.038 to −0.006	−0.011	−0.024 to 0.0009
		WM	−0.007	−0.028 to 0.015	−0.036	−0.056 to −0.015	−0.005	−0.026 to 0.015
Triglycerides (SD)	Global Lipids Genetics Consortium	IVW	−0.0009	−0.013 to 0.012	0.002	−0.013 to 0.017	−0.004	−0.016 to 0.008
		WM	0.011	−0.009 to 0.032	0.019	−0.003 to 0.04	0.003	−0.017 to 0.023

^a^Excluding SNPs related to height (rs724577(*LCORL*) and rs1042725 (*HMGA2*)).

^b^Including SNPs which did not reach genome wide significance for birth weight but p value < 10^−5^ (rs5415 (*SLC2A4*); rs5758511 (*CENPM*); and rs7780752 (*CALCR*)).

HDL: High density lipoprotein; IVW: Inverse variance weighting method; LDL: Low density lipoprotein; WM: Weighted median method.
