# Supplementary material for: Birth weight and risk of ischemic heart disease: A Mendelian randomization study
Source: Sci Rep. 2016 Dec 7;6:38420. doi: 10.1038/srep38420 (PMC5141503; doi:10.1038/srep38420)
Supplement: Supplementary Information [file srep38420-s1.doc]

Supplementary information:

Appendix 1: Characteristics of single nucleotide polymorphisms (SNPs) used in the Mendelian Randomization analysis of the effect of birth weight^1^ on ischemic heart diseases (IHD) and myocardial infarction (MI)^2-5^

|  | GWAS on birth weight | | | CARDIoGRAMplusC4D 1000 Genomes-based GWAS (IHD) | | CARDIoGRAMplusC4D 1000 Genomes-based GWAS (MI) | | CARDIoGRAMplusC4D metabochip/ CARDIoGRAM GWAS^a^ (IHD) | |
| --- | --- | --- | --- | --- | --- | --- | --- | --- | --- |
| SNP | Effect allele | Increase in exposure (z score)  per effect allele  (SE) | P value | Increase in log odds per effect allele  (SE) | P value | Increase in log odds per effect allele  (SE) | P value | Increase in log odds per effect allele  (SE) | P value |
| rs724577 | A | 0.042 (0.0063) | 4.55x10^-11^ | -0.0287 (0.0106) | 0.007 | -0.0403 (0.0118) | 0.0006 | -0.0317 (0.0278) | 0.254 |
| rs900400 | T | 0.072 (0.0056) | 3.58x10^-38^ | -0.0125 (0.0096) | 0.19 | -0.0159 (0.0107) | 0.137 | 0.0273 (0.0169) | 0.106 |
| rs1042725 | C | 0.047 (0.0051) | 1.43x10^-19^ | -0.0204 (0.0093) | 0.028 | -0.0186 (0.0105) | 0.075 | -0.0192 (0.0087) | 0.0276 |
| rs1801253 | C | 0.041 (0.0070) | 3.57x10^-9^ | 0.0146 (0.0106) | 0.17 | 0.0174 (0.0118) | 0.141 | 0.0216 (0.0116) | 0.063 |
| rs4432842 | T | 0.034 (0.0063) | 4.56x10^-8^ | 0.0083 (0.0098) | 0.40 | 0.0102 (0.0109) | 0.351 | -0.0016 (0.0154) | 0.919 |
| rs6931514 | A | 0.05 (0.0057) | 1.50x10^-18^ | -0.0208 (0.0102) | 0.04 | -0.0137 (0.0115) | 0.235 | -0.0150 (0.0103) | 0.145 |
| rs9883204 | T | 0.059 (0.0065) | 5.48x10^-20^ | -0.0112 (0.0112) | 0.32 | -0.0109 (0.0123) | 0.375 | 0.0019 (0.0103) | 0.853 |

^a^Estimates for rs90040 and rs4432842 obtained from CARDIoGRAM

Appendix 2: Funnel plots for the Mendelian randomization estimate from each of the single nucleotide polymorphism (SNP) included in this study

References

1 Horikoshi, M. *et al.* New loci associated with birth weight identify genetic links between intrauterine growth and adult height and metabolism. *Nature genetics* **45**, 76-82, doi:10.1038/ng.2477 (2013).

2 Schunkert, H. *et al.* Large-scale association analysis identifies 13 new susceptibility loci for coronary artery disease. *Nature genetics* **43**, 333-338, doi:10.1038/ng.784 (2011).

3 Coronary Artery Disease Genetics, C. A genome-wide association study in Europeans and South Asians identifies five new loci for coronary artery disease. *Nature genetics* **43**, 339-344, doi:10.1038/ng.782 (2011).

4 Consortium, C. A. D. *et al.* Large-scale association analysis identifies new risk loci for coronary artery disease. *Nature genetics* **45**, 25-33, doi:10.1038/ng.2480 (2013).

5 Nikpay, M. *et al.* A comprehensive 1,000 Genomes-based genome-wide association meta-analysis of coronary artery disease. *Nature genetics* **47**, 1121-1130, doi:10.1038/ng.3396 (2015).
